# Supplementary material for: Bifidobacterium species associated with breastfeeding produce aromatic lactic acids in the infant gut
Source: Nat Microbiol. 2021 Oct 21;6(11):1367–82. doi: 10.1038/s41564-021-00970-4 (PMC8556157; doi:10.1038/s41564-021-00970-4)
Supplement: Supplementary file 1 — Supplementary Tables 1–10, Figs. 1–15 with images of uncropped gels and References. [file 41564_2021_970_MOESM1_ESM.pdf]

nature portfolio

https://doi.org/10.1038/s41564-021-00970-4

Supplementary information

Bifidobacterium species associated with breastfeeding produce aromatic lactic acids in the infant gut

In the format provided by the authors and unedited

Supplementary Information for

Bifidobacterium species associated with breastfeeding produce aromatic lactic acids in the infant gut

Contains:

Supplementary Table 1-10

Supplementary Figures 1-15

Supplementary tables

Supplementary Table 1. Aromatic amino acids and derivatives and their retention time, mass-to-charge ratio (*m/z*) and internal standard.

**Pathway**[1]	**Metabolite**	**Retention time [min]**	***m/z***	**ESI$^+$ ion**	**Internal standard**[2]
Phenylalanine	Phenylalanine	2.6	166.0863	$[M+H]^+$	L-Phenylalanine (ring-d5)
Phenylalanine	Phenyllactic acid	4.7	189.0522	$[M+Na]^+$	L-Phenylalanine (ring-d5)
Phenylalanine	Phenylacetic acid	5.3	137.0597	$[M+H]^+$	L-Phenylalanine (ring-d5)
Phenylalanine	Phenylpropionic acid	5.9	151.0754	$[M+H]^+$	L-Phenylalanine (ring-d5)
Tryptophan	Kynurenine	2.5	209.0921	$[M+H]^+$	L-Tryptophan (indole-d5)
Tryptophan	Tryptophan	3.4	205.0972	$[M+H]^+$	L-Tryptophan (indole-d5)
Tryptophan	Xanthurenic acid	3.6	206.0448	$[M+H]^+$	L-Tryptophan (indole-d5)
Tryptophan	Tryptamine	3.6	161.1073	$[M+H]^+$	L-Tryptophan (indole-d5)
Tryptophan	Kynurenic acid	3.9	190.0499	$[M+H]^+$	L-Tryptophan (indole-d5)
Tryptophan	Indolelactic acid	5.0	206.0812	$[M+H]^+$	Indoleacetic acid (2,2-d2)
Tryptophan	Indolealdehyde	5.2	146.0600	$[M+H]^+$	Indoleacetic acid (2,2-d2)
Tryptophan	Indoleacetic acid	5.3	176.0706	$[M+H]^+$	Indoleacetic acid (2,2-d2)
Tryptophan	Indoleethanol	5.4	162.0913	$[M+H]^+$	Indoleacetic acid (2,2-d2)
Tryptophan	Indolepropionic acid	5.9	190.0863	$[M+H]^+$	Indoleacetic acid (2,2-d2)
Tyrosine	Tyrosine	1.3	182.0812	$[M+H]^+$	L-Tyrosine (ring-d4)
Tyrosine	Tyramine	1.3	138.0913	$[M+H]^+$	L-Tyrosine (ring-d4)
Tyrosine	4-Hydroxyphenyllactic acid	3.5	205.0471	$[M+Na]^+$	L-Tyrosine (ring-d4)
Tyrosine	4-Hydroxyphenylacetic acid	3.9	153.0546	$[M+H]^+$	L-Tyrosine (ring-d4)
Tyrosine	4-Hydroxyphenylpropionic acid	4.6	189.0522	$[M+Na]^+$	L-Tyrosine (ring-d4)
IS	L-Phenylalanine (ring-d5)	2.6	171.1176	$[M+H]^+$	-
IS	L-Tryptophan (indole-d5)	3.4	210.1285	$[M+H]^+$	-
IS	L-Tyrosine (ring-d4)	1.3	186.1063	$[M+H]^+$	-
IS	Indoleacetic acid (2,2-d2)	5.3	178.0832	$[M+H]^+$	-

[1]IS, internal standard

[2]Four different internal standards were used to obtain semi-quantitative concentrations.

Supplementary Table 2. Faecal concentration of aromatic amino acid metabolites in the SKOT cohort (n=59).

Metabolite	Prevalence (%)	Concentration (nmol/g faeces)[1]			
		Median [IQR]	Mean [±SD]	Min	Max
Tyrosine metabolites					
Tyrosine	100.0	372.8 [291.3-503.2]	387.5 [±148.1]	89.9	756.6
Tyramine	91.5	11.2 [2.9-38.8]	45.5 [±90.4]	0.0	447.0
4-hydroxyphenyllactic acid	50.8	1.5 [0.0-9.0]	11.5 [±28.9]	0.0	179.9
4-hydroxyphenylacetic acid	96.6	159.3 [55.0-380.2]	219.6 [±190.1]	0.0	680.9
4-hydroxyphenyl-propionic acid	28.8	0.0 [0.0-11.2]	5.7 [±10.8]	0.0	52.7
Phenylalanine metabolites					
Phenylalanine	100.0	221.5 [189.7-257.4]	222.1 [±56.2]	71.8	360.6
Phenyllactic acid	88.1	16.0 [5.3-31.1]	20.4 [±18.2]	0.0	69.7
Phenylacetic acid	100.0	212.2 [102.0-307.4]	216.1 [±135.7]	3.1	551.7
Phenylpropionic acid	55.9	7.2 [0.0-21.6]	18.1 [±41.0]	0.0	293.1
Tryptophan metabolites					
Tryptophan	100.0	167.6 [123.8-203.9]	160.1 [±60.3]	15.7	275.3
Tryptamine	94.9	4.0 [1.5-9.0]	8.7 [±13.1]	0.0	68.0
Kynurenine	66.1	0.5 [0.0-0.8]	0.4 [±0.4]	0.0	1.0
Kynurenic acid	100.0	3.8 [2.2-5.7]	4.6 [±3.2]	0.2	14.0
Xanthurenic acid	98.3	1.2 [0.9-1.8]	1.4 [±0.9]	0.0	4.3
Indolelactic acid	89.8	17.9 [4.3-44.9]	36.5 [±51.1]	0.0	266.8
Indoleacetic acid	100.0	11.1 [5.2-24.4]	23.6 [±38.6]	1.0	226.0
Indolepropionic acid	67.8	1.5 [0.0-10.0]	6.5 [±9.9]	0.0	49.6
Indolealdehyde	100.0	1.7 [1.4-2.5]	2.1 [±1.2]	0.9	6.9
Indoleethanol	69.5	0.7 [0.0-1.0]	0.7 [±0.6]	0.0	3.0

[1]Semi-quantitative concentrations were obtained using four isotope-labelled internal standards.

Supplementary Table 3. Identification of type 4 *ldh* in whole genome sequenced human gut derived *Bifidobacterium* species.

Species	Number of strains	Number of strains with type 4 *ldh*	Percent of strains with type 4 *ldh*
B. breve	44	44	100 %
B. longum	27	27	100 %
B. animalis	24	0	0 %
B. bifidum	9	9	100 %
B. adolescentis	8	0	0 %
B. kashiwanohense	3	0	0 %
B. pseudolongum	3	0	0 %
B. angulatum	2	2	100 %
B. catenulatum	2	0	0 %
B. dentium	2	0	0 %
B. pseudocatenulatum	2	0	0 %
B. scardovii	1	1	100 %

Supplementary Table 4. Faecal concentration of aromatic amino acid metabolites in the Copenhagen Infant Gut cohort (n=267).

Metabolite	Prevalence (%)	Concentration (nmol/g faeces)[1]			
		Median [IQR]	Mean [±SD]	Min	Max
Tyrosine metabolites					
Tyrosine	100.0	307.4 [194.9-457.9]	358.1 [±231.8]	26.6	1776.0
Tyramine	63.7	11.0 [0.0-194]	185.1 [±363.7]	0.0	2428.0
4-hydroxyphenyllactic acid	93.3	40.6 [10.9-76.6]	53.5 [±54.3]	0.0	292.1
4-hydroxyphenylacetic acid	44.9	0.0 [0.0-41.9]	51.7 [±125.7]	0.0	995.3
4-hydroxyphenyl-propionic acid	16.1	0.0 [0.0-0.0]	13.5 [±64.6]	0.0	733.2
Phenylalanine metabolites					
Phenylalanine	100.0	381.1 [267-497.4]	419.9 [±250.0]	37.1	2159.0
Phenyllactic acid	97.8	38 [17.6-73.4]	54.9 [±56.7]	0.0	477.4
Phenylacetic acid	29.2	0.0 [0.0-9.6]	24.5 [±76.6]	0.0	718.2
Phenylpropionic acid	10.1	0.0 [0.0-0.0]	10.9 [±49.1]	0.0	601.6
Tryptophan metabolites					
Tryptophan	100.0	128.3 [84.6-197.1]	155.7 [±104.0]	10.6	647.8
Tryptamine	25.8	0.0 [0.0-0.1]	4.0 [±13.2]	0.0	104.2
Kynurenine	82.4	0.3 [0.1-0.5]	0.4 [±0.4]	0.0	2.3
Kynurenic acid	100.0	5.9 [3.4-9.0]	8.1 [±8.7]	0.9	90.8
Xanthurenic acid	98.5	0.7 [0.5-1.2]	1.0 [±0.9]	0.0	6.3
Indolelactic acid	98.1	47.7 [15.0-103.4]	69.0 [±70.2]	0.0	332.2
Indoleacetic acid	47.2	0.0 [0.0-1.0]	2.1 [±9.9]	0.0	127.2
Indolepropionic acid	7.1	0.0 [0.0-0.0]	0.5 [±3.2]	0.0	39.0
Indolealdehyde	85.4	0.9 [0.5-2.3]	2.1 [±3.4]	0.0	23.0
Indoleethanol	4.5	0.0 [0.0-0.0]	0.1 [±0.3]	0.0	3.2

[1]Semi-quantitative concentrations were obtained using four isotope-labelled internal standards.

Supplementary Table 5. Primers used for 16S rRNA amplicon sequencing and quantitative PCR.

Primer target	Primer name	Sequence (5’-3’)	Final conc. (μM)	Annealing temperature (°C)	Reference
**Universal bacteria (NGS PCR)**	PBU_X	A-adapter-TCAG-barcode-CCTACGGGAGGCAGCAG	1	72	Laursen et al., 2017[1]
	PBR_P1	trP1-adapter-ATTACCGCGGCTGCTGG	1		
**Universal bacteria (qPCR)**	PBU	CCTACGGGAGGCAGCAG	0.2	60	Tulstrup *et al.*, 2015[2]
	PBR	ATTACCGCGGCTGCTGG	0.2		
B. longum* subsp. *infantis	Blon0915F	CGTATTGGCTTTGTACGCATTT	0.75	50	Frese *et al.*, 2017[3]
	Blon0915R	ATCGTGCCGGTGAGATTTAC	0.75		
B. longum* subsp. *longum	lon_0274_F	GAGGCGATGGTCTGGAAGTT	0.75	50	Lawley *et al.*, 2017[4]
	lon_0274_R	CCACATCGCCGAGAAGATTC	0.75		
B. bifidum	BiBIF-1	CCACATGATCGCATGTGATTG	0.5	60	Matsuki *et al.*, 2004[5]
	BiBIF-2	CCGAAGGCTTGCTCCCAAA	0.5		
B. breve	B_bre-f	GCTCGTCGTTGCCGCCAAGGACGTT	0.5	72	Junick and Blaut, 2012[6]
	B_bre-r	ACAGAATGTACGGATCCTCGAGCACG	0.5		

Supplementary Table 6. *Bifidobacterium* strains used in this study.

Strain	Culture Collection[1]
B. adolescentis E194a	DSM 20083^T
B. animalis subsp. *lactis* UR1	DSM 10140^T
B. animalis subsp. *animalis* R101-8	DSM 20104^T
B. bifidum Ti	DSM 20456^T
B. breve S1	DSM 20213^T
B. dentium B764	DSM 20436^T
B. longum subsp. *longum* E194b	DSM 20219^T
B. longum subsp*. longum* 105-A	JCM 31944
B. longum subsp. *longum* 105-A type 4 *ldh*::pMSK127	-
B. longum subsp. *longum* 105-A type 4 *ldh*::pMSK127 / pMSK128 (P*xfp*-type4 *ldh*)	-
B. longum subsp*. infantis* S12	DSM 20088^T
B. pseudolongum subsp*. pseudolongum* PNC-2-9G	DSM 20099^T
B. scardovii CCUG 13008 A	DSM 13734^T
B. catenulatum B669	DSM 16992^T
B. pseudocatenulatum B1279	DSM 20438^T

[1]Superscript “T” indicates type strain

Supplementary Table 7. Primers used for generation of type 4 *ldh* insertional mutant and type 4 *ldh* complemented strain.

Primer name	Primer sequence (5′-nucleotide sequence-3′)[1]
Pr-543	TATATATGAGTACTGaggtgcagcgcgtgctcctt
Pr-546	CAGGCATGCAAGCTTtggcctacgaacctgtctgg
Pr-580	CCAGCTCAAGGGATCgcccgcgacctcgatgatg
Pr-581	CGGTACCCGGGGATCgtggacacggaggcgaaac
Pr-598	GGAAGATCACTTCGCttcgaacatgggaaatcgaa
Pr-599	GTTCATAGTGACCATaagcactccttgggggccgc
Pr-600	atggtcactatgaaccgcaacaaag
Pr-601	CGTGGGATCCGTCGAtcacagcagcccctcgcagt

[1]Uppercase letter indicates sequence for In-Fusion cloning

Supplementary Table 8. Human milk oligosaccharides (HMOs), their retention time and mass-to-charge ratio (*m/z*).

**Abbreviation**	**HMO**	**Retention time [min]**	**[*m/z*]**	**ESI$^+$ ion**
2’FL / 3FL	2'-*O*-Fucosyllactose / 3-*O*-Fucosyllactose	0.6	489.1814	$[M+H]^+$
LNT / LN*n*T	Lacto-*N*-tetraose / Lacto-*N*-*neo*tetraose	0.7	708.2557	$[M+H]^+$
3’SL / 6’SL	3'-*O*-Sialyllactose / 6'-*O*-Sialyllactose	0.7	634.2189	$[M+H]^+$

Supplementary Table 9. Optimized parameters used for pre-processing the urine metabolome data in mzMine2.8

**Batch step**		**Parameters**
Negative Mode	Mass detection	Noise level: 1.0E1
	Chromatogram builder	Min time span (min): 0.01<br>Min height:3.0E1 ; *m/z* tolerance: 0.06 mz or 25 ppm
	Chromatogram deconvolution	Chromatographic threshold: 90%; Search minimum in RT range (min): 0.01; Minimum relative height: 5%; Minimum absolute height: 3.0E1; Min ratio of peak/top edge: 1.0; Peak duration range (min): 0.01-0.2
	Isotopic pattern	*m/z* tolerance: 0.06 or 30 ppm; Retention time tolerance: 0.02; Monotonic shape; maximum charge: 1
	Join aligner	*m/z* tolerance: 0.03 or 20 ppm; Absolute retention time tolerance: 0.2; Weight for both *m/z* tolerance and retention time tolerance: 10
	Peak finder (gap filling)	Intensity tolerance: 50%; *m/z* tolerance: 0.03or 20 ppm; Absolute retention time tolerance: 0.03

**m/z*: mass to charge ratio; RT: retention time; Intensity: Peak area

Supplementary Table 10. Reagents used for human T-cell cultures.

Reagent	Source	Cat no. / RRID
Triton X-100	Sigma-Aldrich	T8787
Paraformaldehyde (PFA)	Sigma-Aldrich	P6148
Low melting point agarose	Invitrogen	16520-100
Fetal Bovine Serum (FBS)	Gibco	ADD
4´,6´-diamidino-2-phenylindole (DAPI)	Thermo Fisher	D1306
Tween-20	Sigma-Aldrich	P1379
Gibco™ DPBS	Thermo Fisher	14-190-144
ß-mercopatoethanol		
Hepes	Gibco	
L-glutamine	Gibco	
Penicillin / Streptomycin	Thermo Fisher	15140122
H_2SO_4	Sigma-Aldrich	339741
IL-6	R&D	206-IL-050
TGFβ	R&D	240-B-010
IL-23	R&D	1290-IL-010
IL-1β	R&D	201-LB-010
ImmunoCult™ Human CD3/CD28 T Cell Activator	Stemcell	10971
Lymphoprep	Axis Shield Poc As	11508545

Supplementary figures

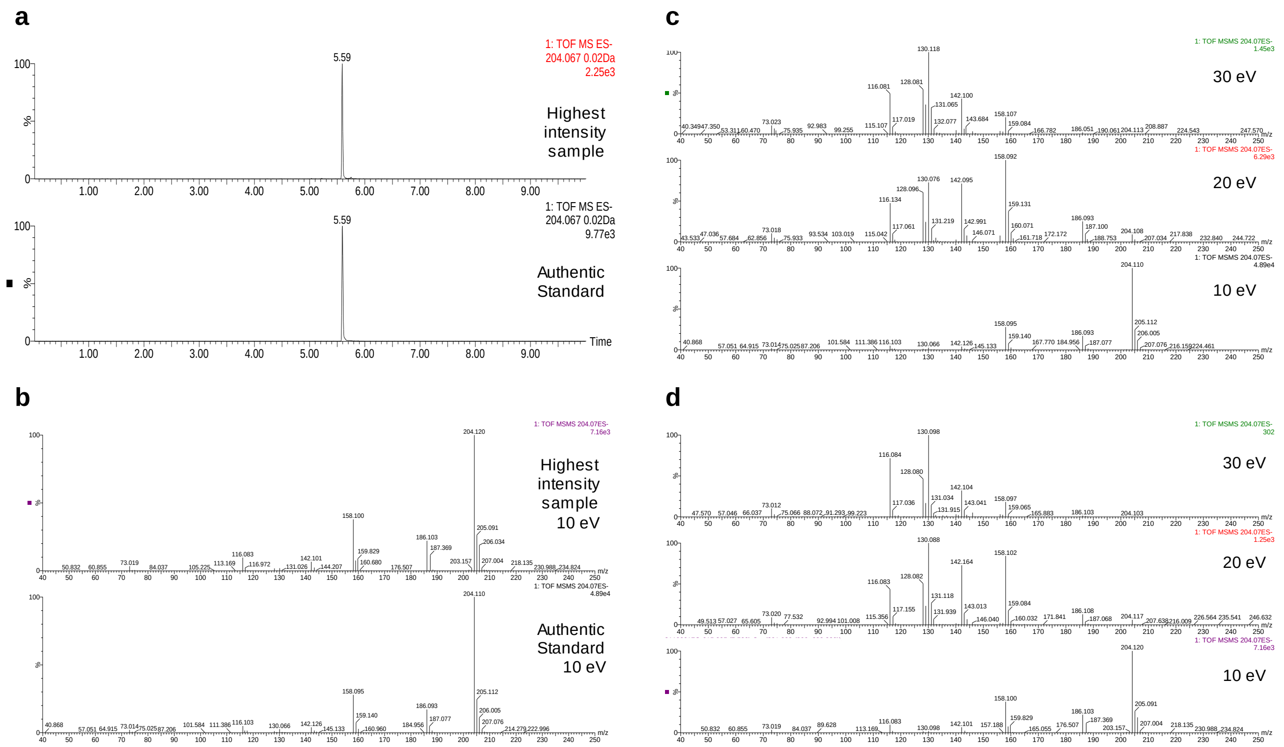

Supplementary Figure 1. Identification of indolelactic acid in urine by extracted ion chromatograms and fragmentation profiles of indolelactic acid and sample with the highest intensity.

a, Feature defined with 204.067 *m/z* and 5.59 RT in negative ionization mode confirmed with authentic standard of indole lactic acid. **b**, MS/MS fragmentation profile of authentic standard and the highest intensity sample fragmented at 10 eV collision dissociation energy. **c**, MS/MS fragmentation profile of authentic standard of indole lactic acid at 10, 20 and 30 eV collision dissociation energies. **d**, MS/MS fragmentation profile of sample with highest intensity at 10, 20 and 30 eV collision dissociation energies.

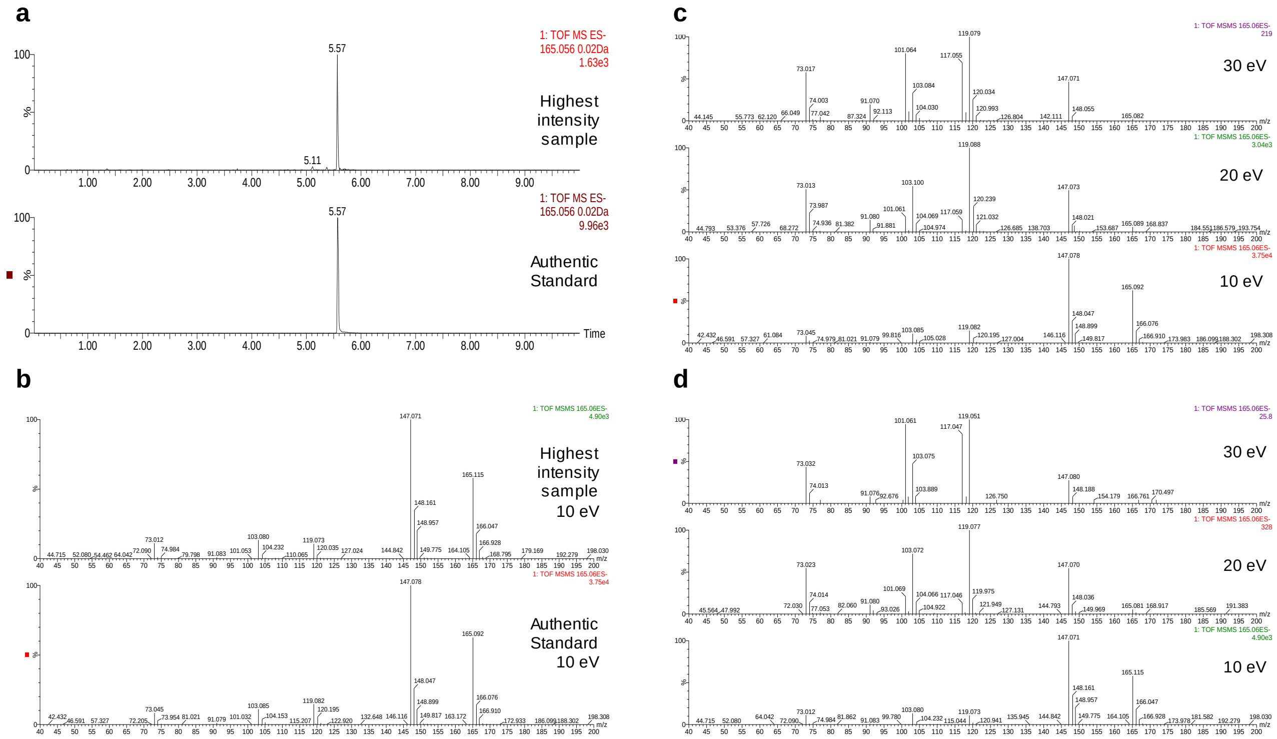

Supplementary Figure 2. Identification of phenyllactic acid in urine by extracted ion chromatograms and fragmentation profiles of phenyllactic acid and sample with the highest intensity.

a, Feature defined with 165.056 *m/z* and 5.57 RT in negative ionization mode confirmed with authentic standard of phenyl lactic acid. **b**, MS/MS fragmentation profile of authentic standard and the highest intensity sample fragmented at 10 eV collision dissociation energy. **c,** MS/MS fragmentation profile of authentic standard of phenyl lactic acid at 10, 20 and 30 eV collision dissociation energies. **d**, MS/MS fragmentation profile of sample with highest intensity at 10, 20 and 30 eV collision dissociation energies.

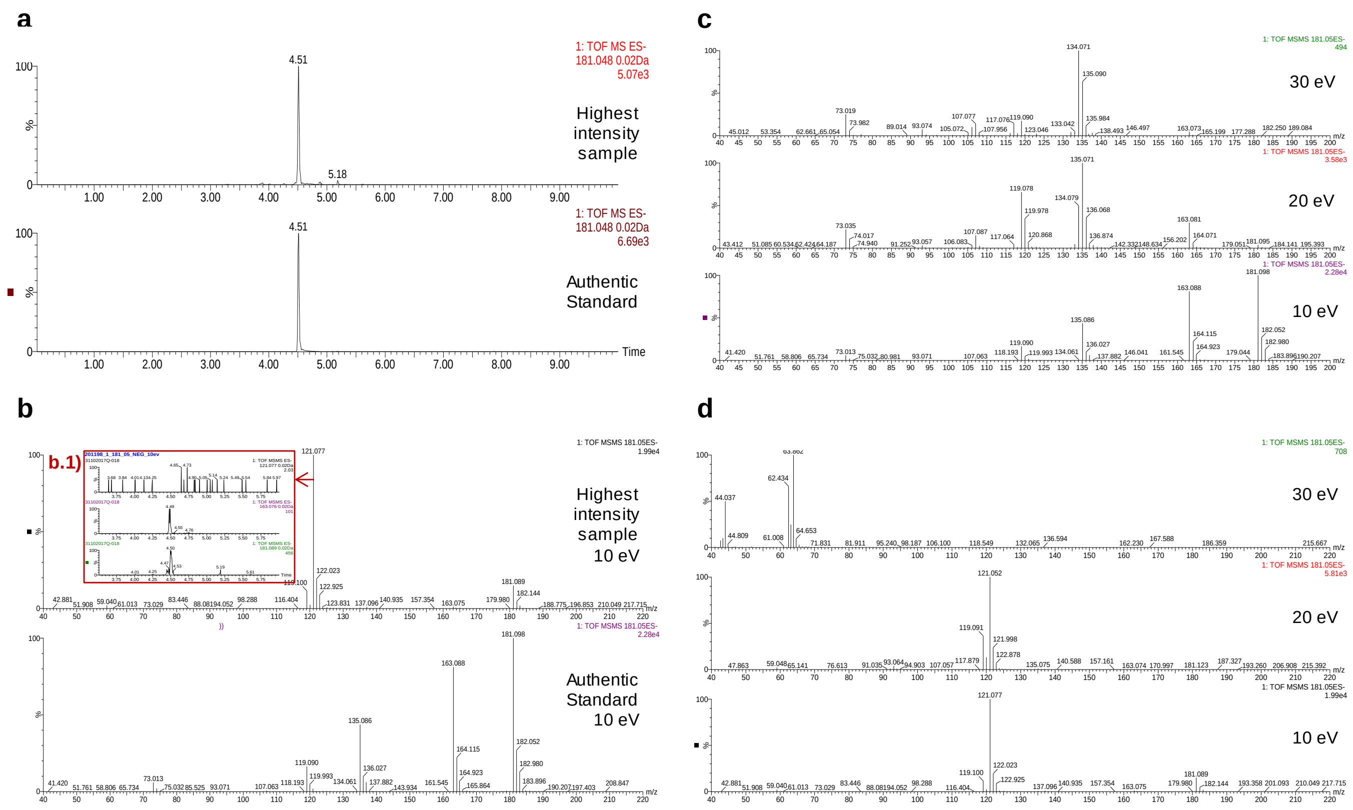

Supplementary Figure 3. Identification of 4-hydroxyphenyllactic acid in urine by extracted ion chromatograms (EICs) and fragmentation profiles of 4-hydroxyphenyllactic acid and sample with the highest intensity.

a, Feature defined with 181.048 *m/z* and 4.51 RT in negative ionization mode confirmed with authentic standard of 4-hydroxyphenyllactic acid. **b**, MS/MS fragmentation profile of authentic standard and the highest intensity sample fragmented at 10 eV collision dissociation energy. b.1) EIC of 181.089 *m/z* (parent ion), 163.075 *m/z* (main fragment) and 121.077 *m/z* shown with arrow indicates that the feature with 121.077 *m/z* does not belong to the peak of 4-hydroxyphenyllactic acid. **c**, MS/MS fragmentation profile of authentic standard of 4-hydroxyphenyllactic acid at 10, 20 and 30 eV collision dissociation energies. **d**, MS/MS fragmentation profile of sample with highest intensity at 10, 20 and 30 eV collision dissociation energies.

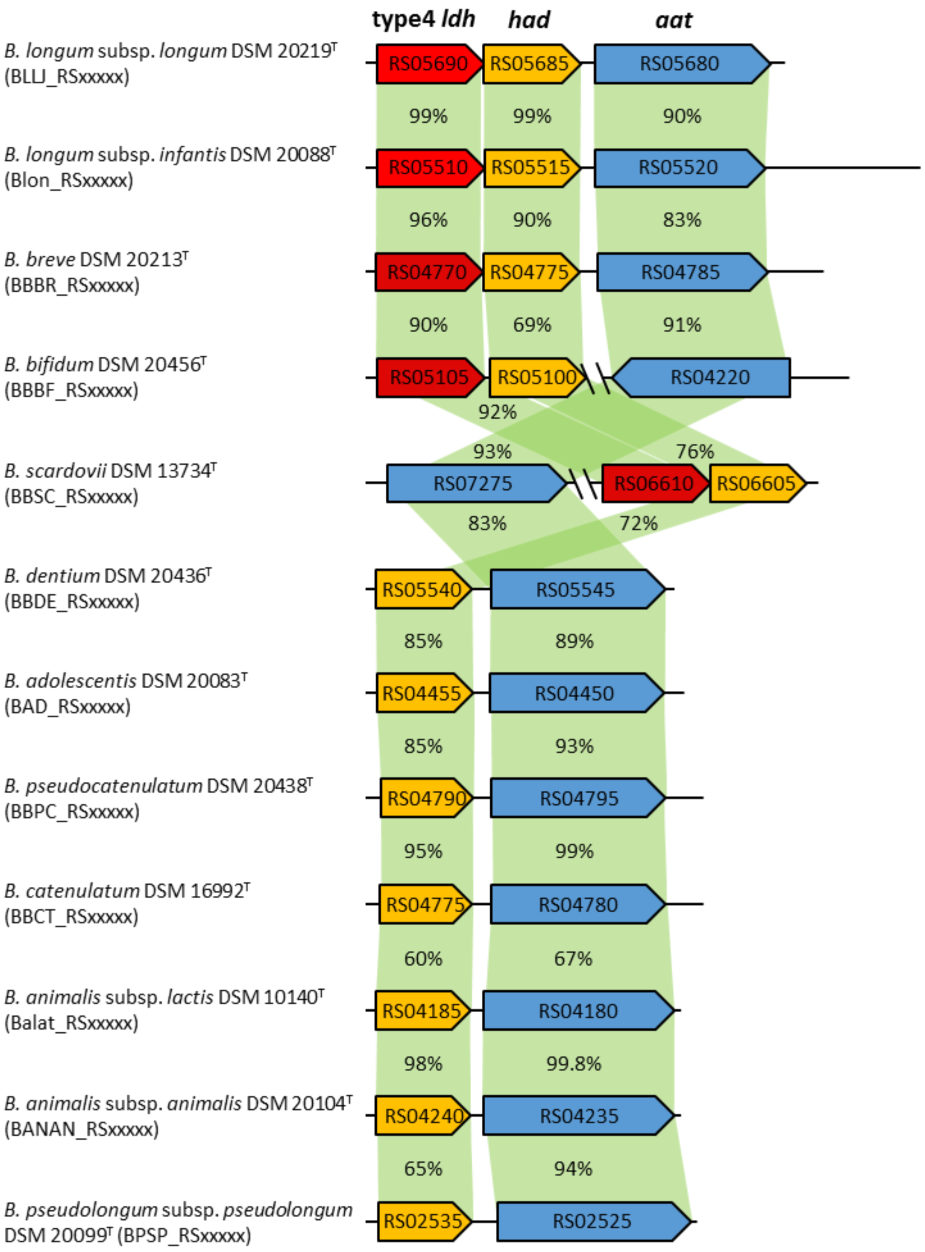

Supplementary Figure 4. Comparison of 'type 4' *ldh* gene cluster in human-gut-associated *Bifidobacterium* type strains. The 'type 4' *ldh*, *had*, and *aat* genes are represented by red, yellow, and blue arrows, respectively. The amino acid sequence identity was determined by conducting pairwise alignments in MBGD (Microbial Genome Database for Comparative Analysis; http://mbgd.genome.ad.jp/). *ldh*: lactate dehydrogenase gene, *had*: haloacid dehalogenase gene, *aat*: amino acid transaminase gene. The gene organization was drawn by drawGeneArrows3 (http://www.ige.tohoku.ac.jp/joho/labhome/tool.html).

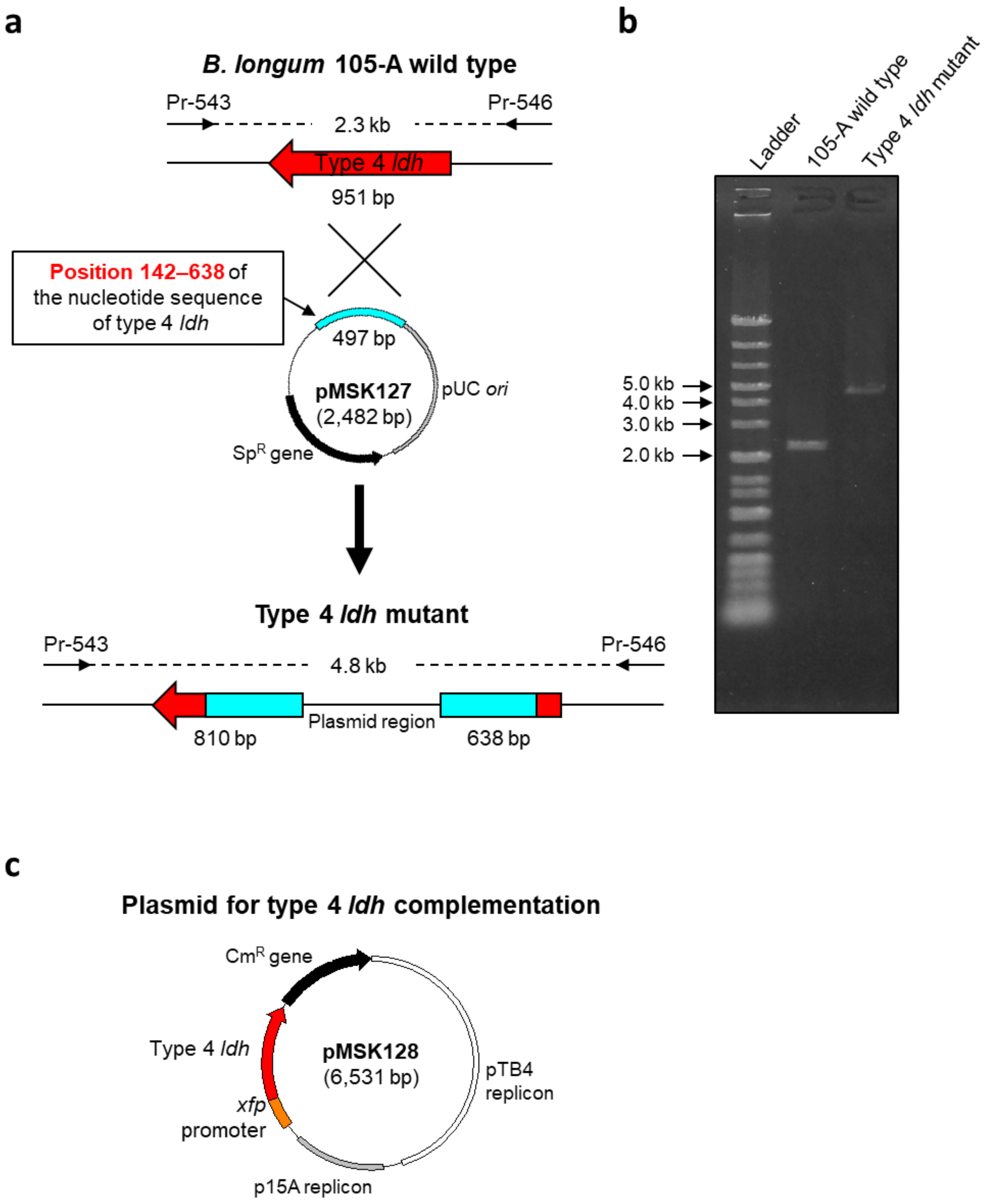

Supplementary Figure 5. Generation of the type 4 *ldh* insertional mutant and type 4 *ldh* complemented strain. a, Generation of type 4 *ldh* insertional mutant by single cross-over recombination with pMSK127 carrying the partial *ldh* gene from *B. longum* subsp. *longum* 105-A (BL105A_0985). **b**, Insertion of pMSK127 was verified by genomic PCR using primers (Pr-543/Pr-546) designed to anneal the flanking regions of the *ldh* gene. The analysis was performed once. **c**, pMSK128, which carries the *ldh* gene (BL105A_0985) under the control of *xfp* promoter, was introduced into type 4 *ldh* mutant to generate the type 4 *ldh*-complemented strain.

a

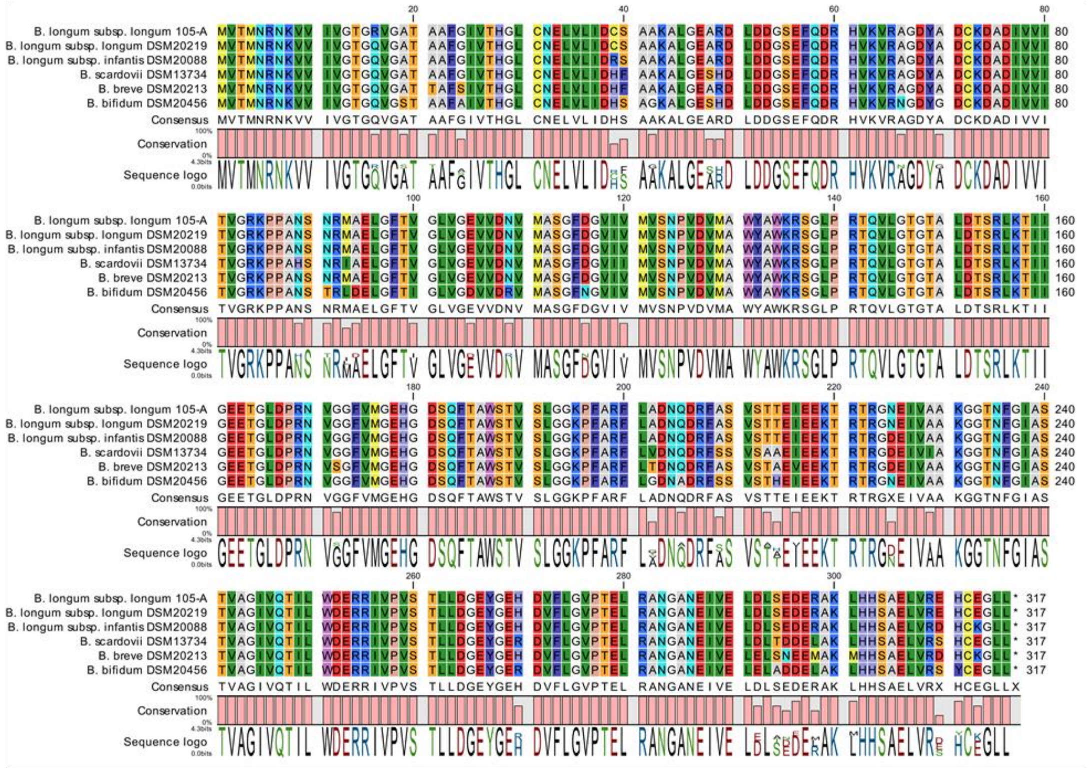

b

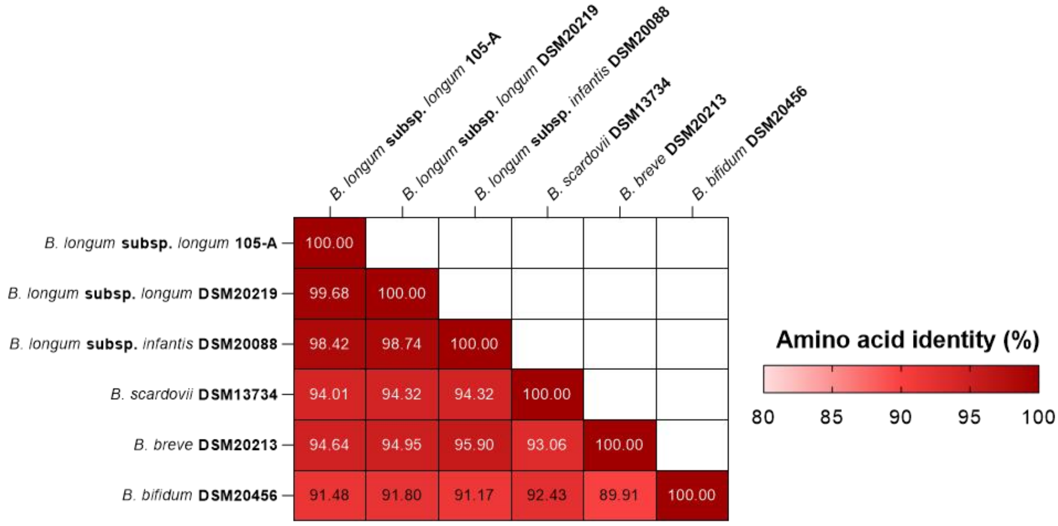

Supplementary Figure 6. Analysis of type 4 LDH amino acid sequences.

a, Alignment of the type 4 LDH amino acid sequences of *B. longum* subsp. *longum* 105-A and type strains of *B. longum* subsp. *longum*, *B. longum* subsp. *infantis*, *B. scardovii*, *B. breve* and *B. bifidum*. **b,** Heatmap showing amino acid percent identities in pairwise comparisons between the six strains.

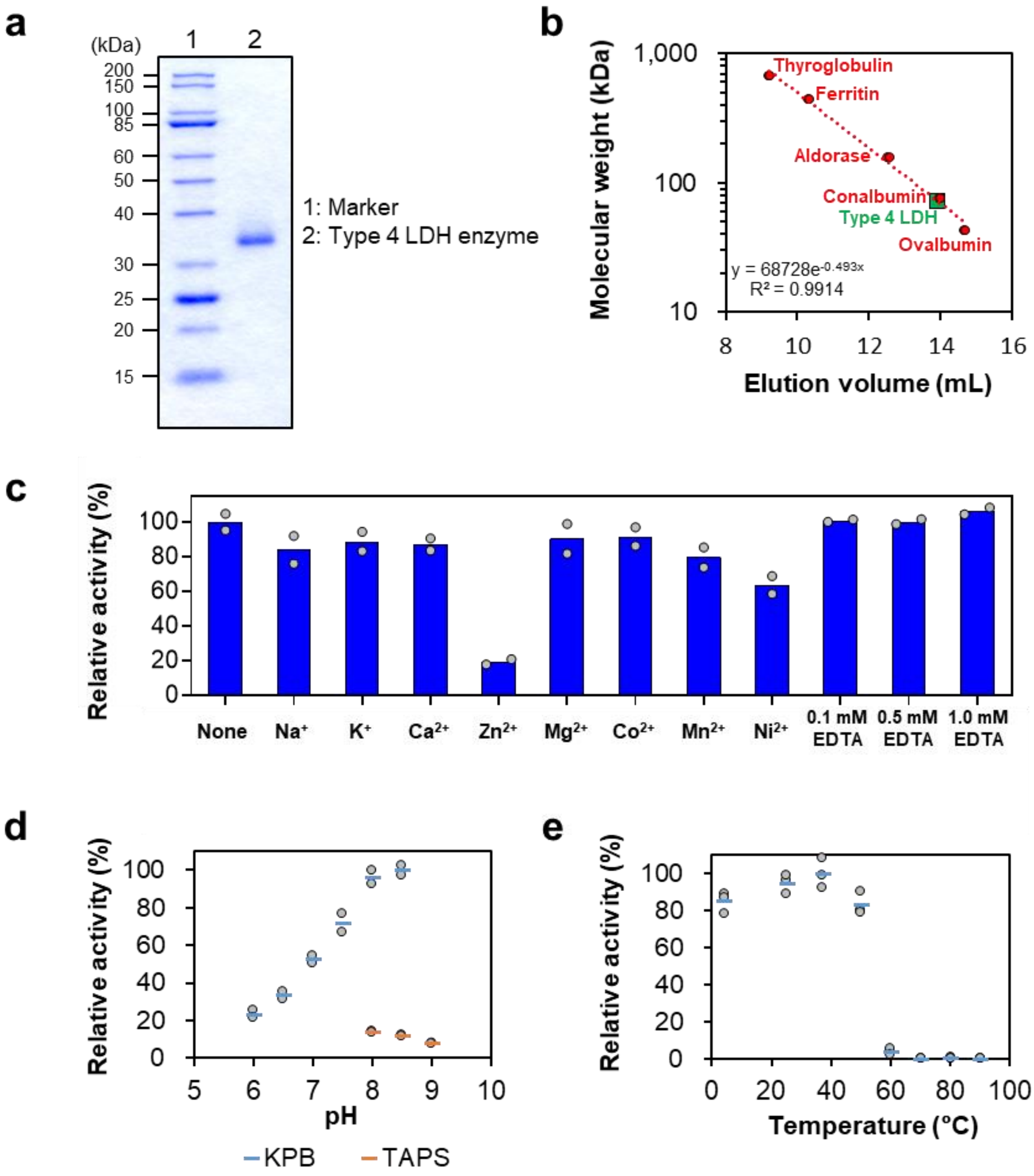

Supplementary Figure 7. The physicochemical properties of type 4 LDH enzyme.

a, Representative picture of two independent SDS-PAGE runs of type 4 LDH enzyme. Lane 1, Marker; Lane 2, purified type 4 LDH enzyme. **b**, The native molecular mass of the recombinant type 4 LDH was estimated by size exclusion chromatography. Thyroglobulin (669 kDa), ferritin (440 kDa), aldolase (158 kDa), conalbumin (75 kDa), ovalbumin (43 kDa) were used as molecular markers. Symbols (dots for markers and squares for type 4 LDH) indicate the values obtained for two independent assays. **c**, The effect of metal ions on activity. Dots and bars indicate the respective values and means of two independent assays, respectively. **d**, The effect of pH on activity. Data are shown by dots and lines representing the values of two independent assays and the means, respectively. **e**, The temperature stability of the enzyme. Data are shown by dots and lines that indicate the values of three independent assays and the means, respectively.

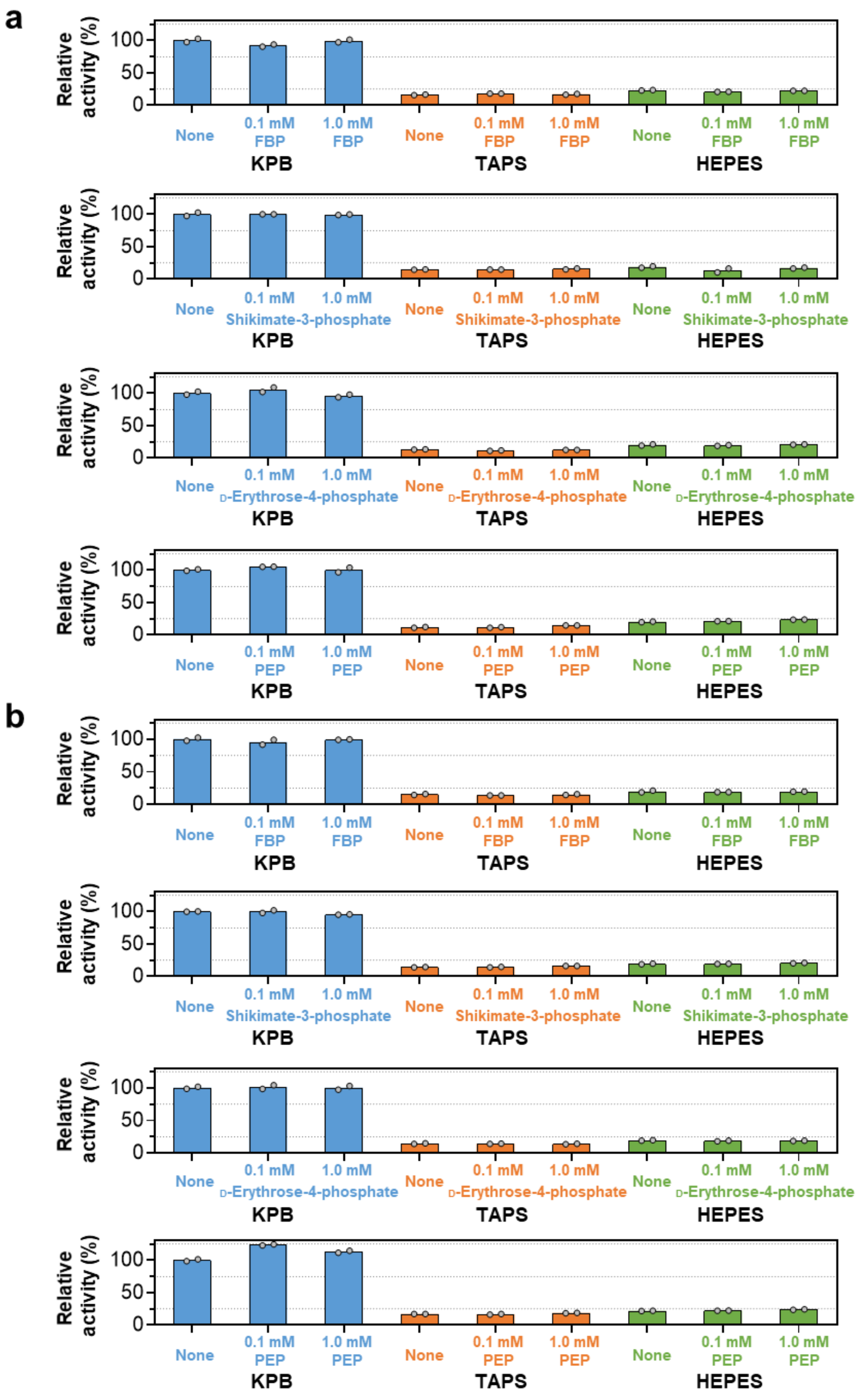

Supplementary Figure 8. The effect of phosphate compounds on the activity of type 4 LDH enzyme. a-b, The compounds with phosphate group were added into the reaction mixture containing either 50 mM KPB, TAPS buffer, or HEPES buffer (pH 8.0) prior to the enzymatic assay. Substrate phenylpyruvic acid was added to give a final concentration of 1 mM (**a**) and 4 mM (**b**). FBP, fructose-1,6-bisphosphate; PEP, phosphoenolpyruvic acid. Data are shown with dots and bars that represent the values obtained for two independent assays and the means, respectively.

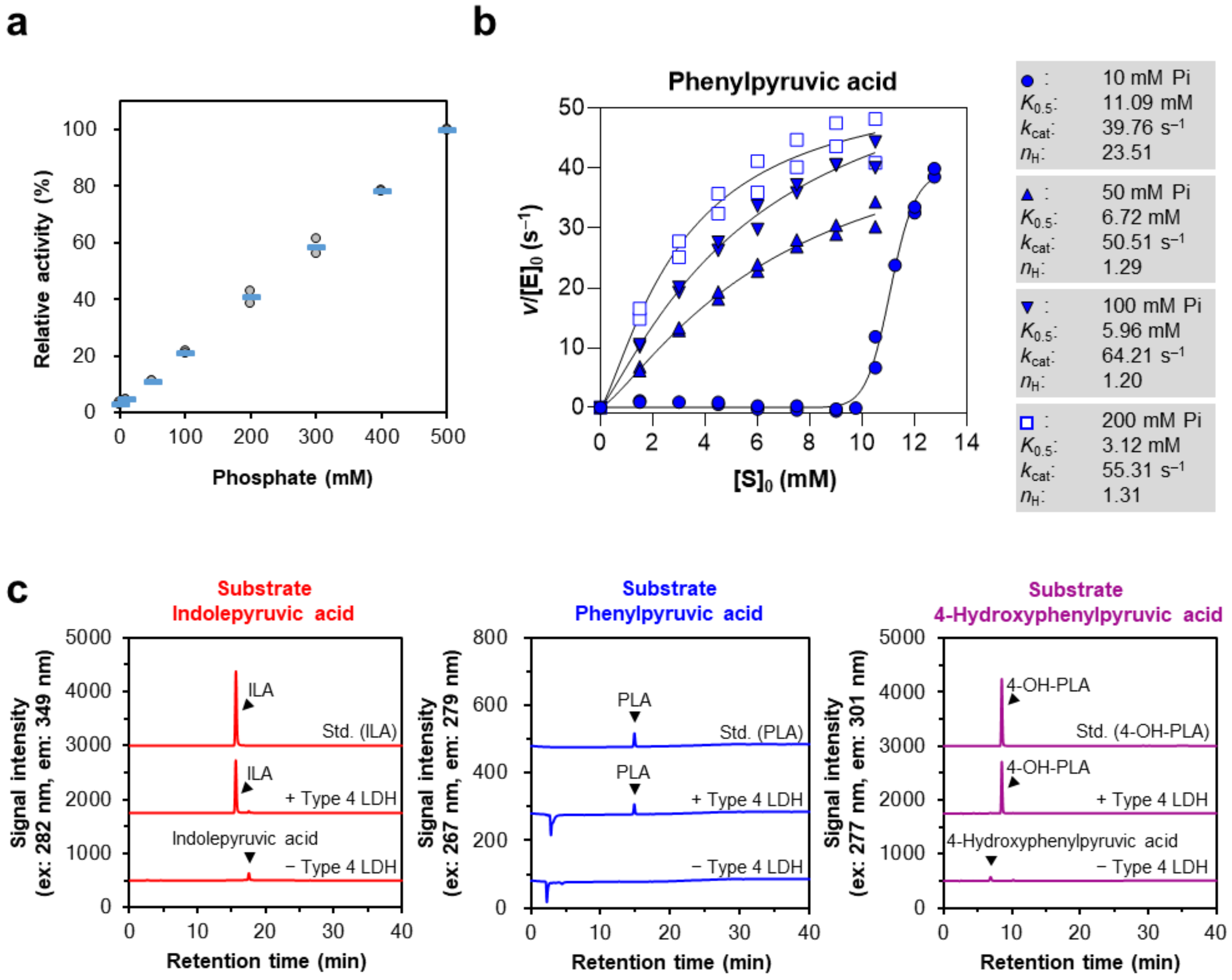

Supplementary Figure 9. Effect of phosphate concentration on type 4 LDH activity and HPLC chromatograms showing formation of aromatic lactic acids from respective aromatic pyruvic acids.
a, The effect of phosphate ion on enzyme activity. Data are shown with dots and lines representing the values obtained for two independent assays and the means, respectively. **b**, Substrate saturation curves obtained for type 4 LDH in the presence of different concentrations of phosphate. Phenylpyruvic acid was used as a substrate. The parameters were calculated by curve-fitting two independent experimental data to Hill equation. The same data as in Fig. 3b was used for 100 mM Pi. **c**, Formation of aromatic lactic acids from respective aromatic pyruvic acids, verified by HPLC analysis. The reaction mixture contained 100 mM KPB (pH 8.0), 1mM 2-ME, 1 mM β-NADH, 1.5 µM type 4 LDH enzyme, and 1 mM substrate. After incubation at 37 °C for 30 min, the reaction mixtures were analysed by HPLC. Ten-fold dilution was applied before HPLC run for the samples containing indolepyruvic acid as the substrate. Standard concentrations were 0.1 mM (ILA) or 1 mM (PLA and 4-OH-PLA). Note that phenylpyruvic acid was not detected under the used excitation/detection conditions. One representative chromatogram of two independent runs for each compound is shown.

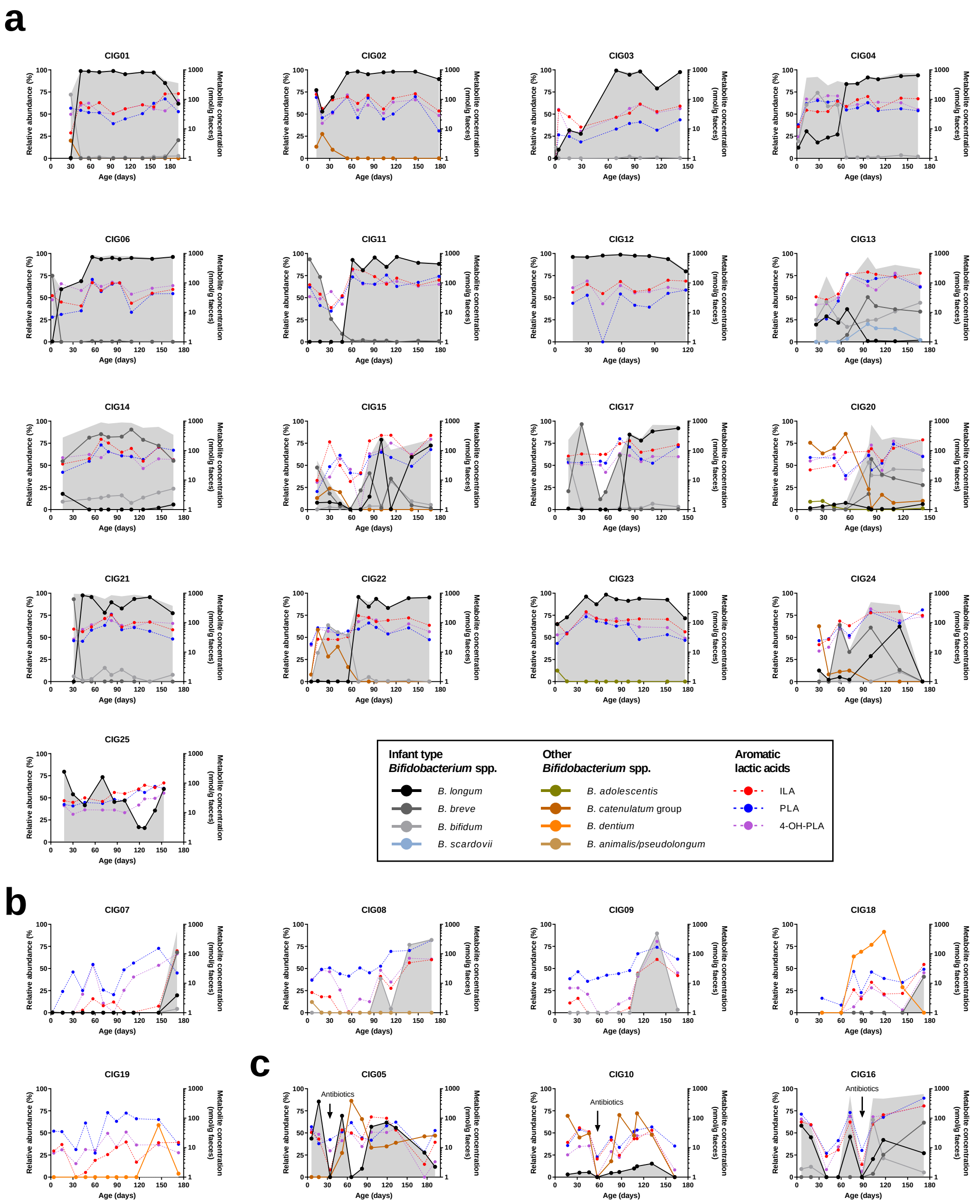

Supplementary Figure 10. Relative abundances of *Bifidobacterium* species and concentrations of aromatic lactic acids in the CIG cohort.

Relative abundance of *Bifidobacterium* spp. (average relative abundance >1% of total community) and concentrations of indolelactic acid (ILA), phenyllactic acid (PLA) and 4-hydroxyphenyllactic acid (4-OH-PLA) in all individuals from the Copenhagen Infant Gut (CIG) cohort. Values of metabolite concentrations below 1 nmol/g faeces are not shown. Infant type *Bifidobacterium* spp. is the sum of the absolute abundances of *B. longum*, *B. breve*, *B. bifidum* and *B. scardovii* and is indicated with grey background shading. **a**, Infants early and consistently colonised with infant type *Bifidobacterium* spp. (colonize within first month reaching average relative abundance >40% during first 6 months). **b**, Infants with late colonization of infant type *Bifidobacterium* spp. (not detectable or on average <0.5% of total community within the first 3 months of life) and **c**, Infants that received oral antibiotics during sampling.

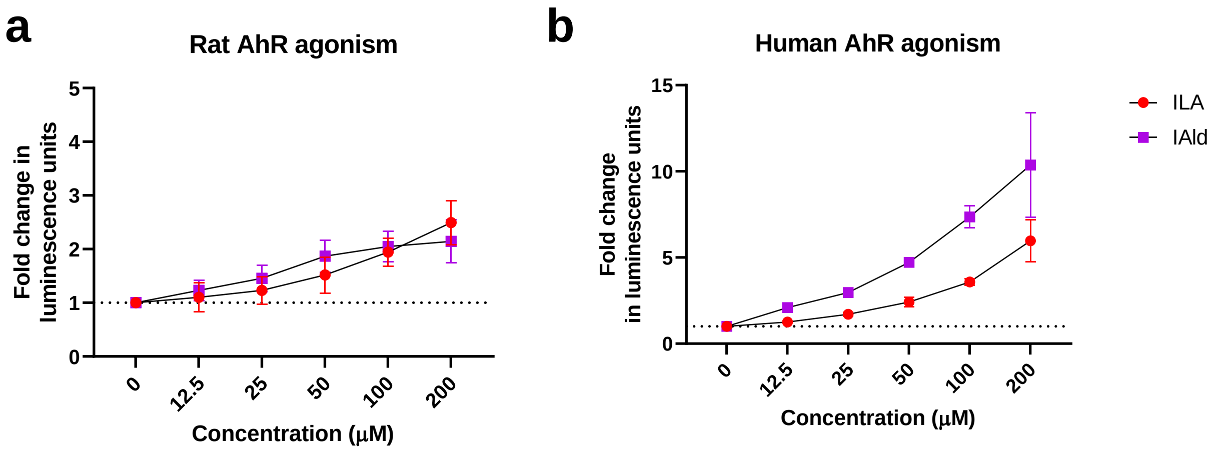

Supplementary Figure 11. Indolelactic acid is an aryl hydrocarbon receptor (AhR) agonist. Dose-dependent effects of indolelactic acid (ILA) and indolealdehyde (IAld) in **a,** rat and **b,** human AhR reporter assays. The AhR activity (Luminescence Units) of the indoles were reported as fold changes relative to vehicle signal (0 µM). Data are presented as mean+SD of two (**a**) to three (**b**) experiments including three technical replicates.

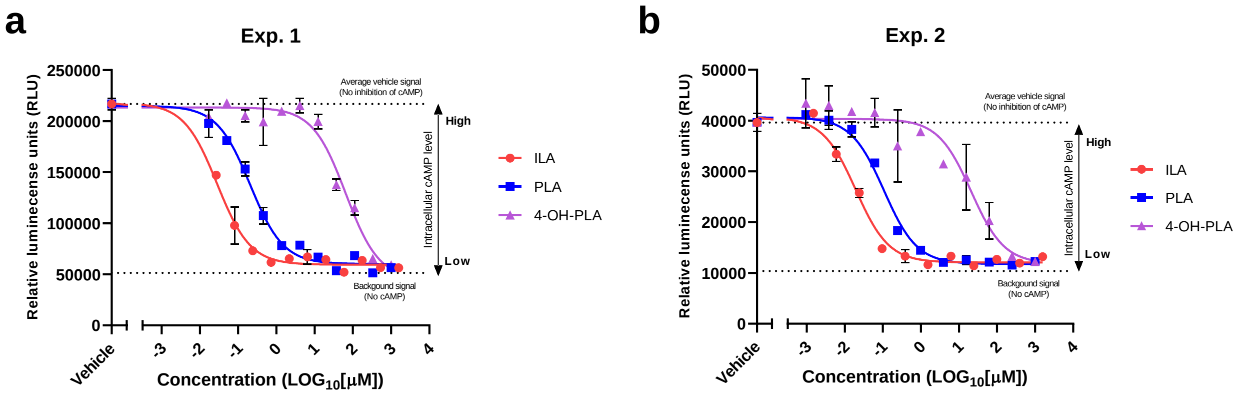

Supplementary Figure 12. Aromatic lactic acids are hydroxycarboxylic acid 3 (HCA_3) receptor agonist.

Dose-dependent effects of indolelactic acid (ILA), phenyllactic acid (PLA) and 4-hydroxyphenyllactic acid (4-OH-PLA) in a human HCA_3 receptor assay (cAMP Hunter™ eXpress GPR109B CHO-K1 GPCR Assay). Activation of the G-protein coupled HCA_3 receptor results in activation of the Gα-inhibitory subunit, which inhibits intracellular adenylyl cyclase activity and reduces forskolin-induced cAMP levels. cAMP levels are detected by a competitive immunoassay based on β-galactosidase hydrolysis of a substrate producing a luminescence signal. The ability of the aromatic lactic acids to activate the HCA_3 receptor results in a decrease of forskolin-induced cAMP levels, resulting in inhibition of the luminescence signal (Relative Luminescence Units). Serial dilutions of the aromatic lactic acids were compared to average vehicle signal (0 µM) and average background signal (no forskolin induction of cAMP). Data are presented as two independent experiments (**a** and **b**) with mean±SD of two technical replicates each. Curves were fitted to the data points by non-linear regression.

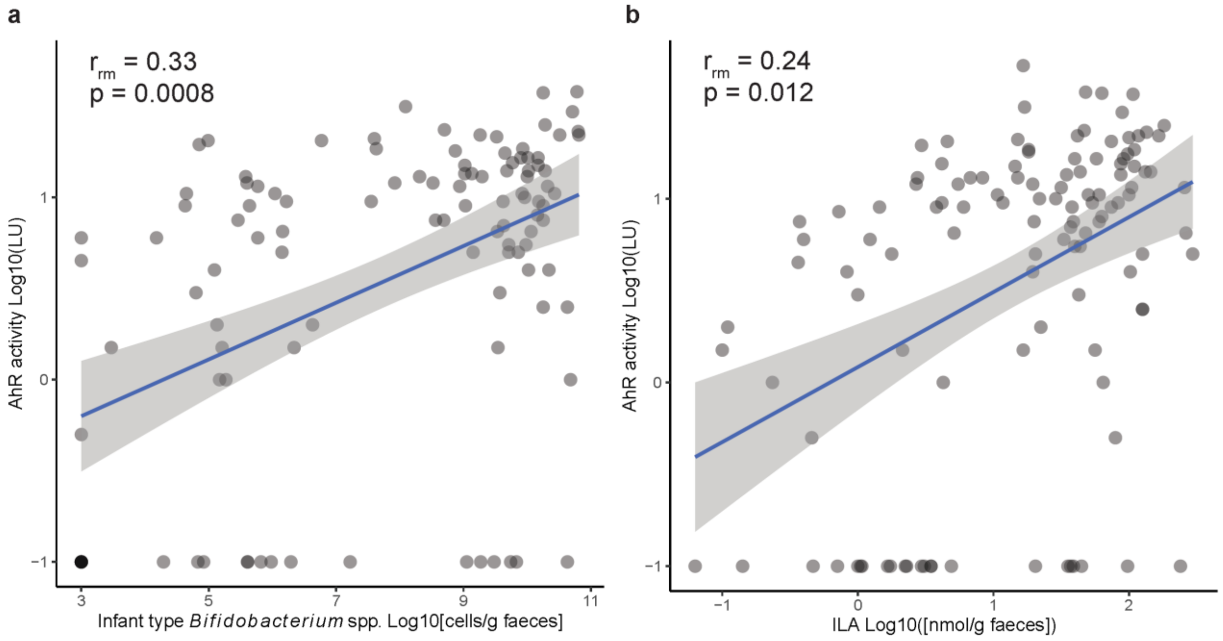

Supplementary Figure 13. Associations between Infant type *Bifidobacterium* species/ILA and AhR activity in faecal water from the CIG infants.
Scatter dot plots of the relationship between faecal abundance of **a**, infant type *Bifidobacterium* spp or **b**, ILA and AhR activity (Luminescence Units, LU) measured in faecal water of the CIG infants. Statistical significance is evaluated by two-sided repeated measures correlations (r_{rm} is the repeated measures correlation coefficient). Linear regression curve fits are shown with coloured mean line and 95% CI indicated in grey shading.

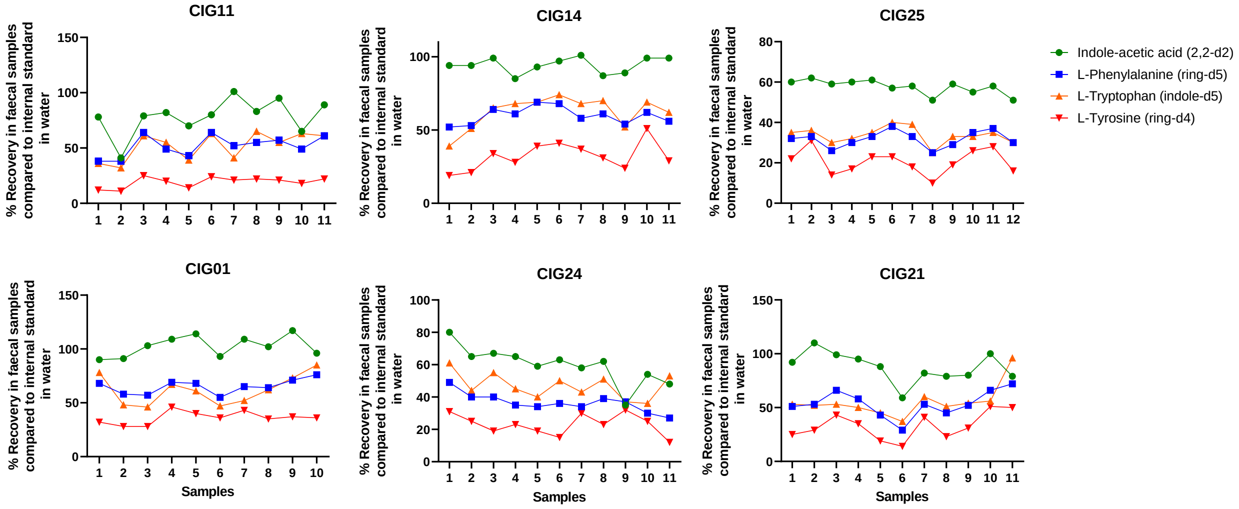

Supplementary Fig 14. Recoveries of internal standards in stool samples.
The recovery of internal standards in stool samples varied. However, the recovery of the internal standards relative to each other were in general rather consistent as demonstrated by the relative recoveries (Internal standard peak area in faecal sample divided by internal standard peak area in water) of internal standards in stool samples of six Copenhagen Infant Gut (CIG) infants.

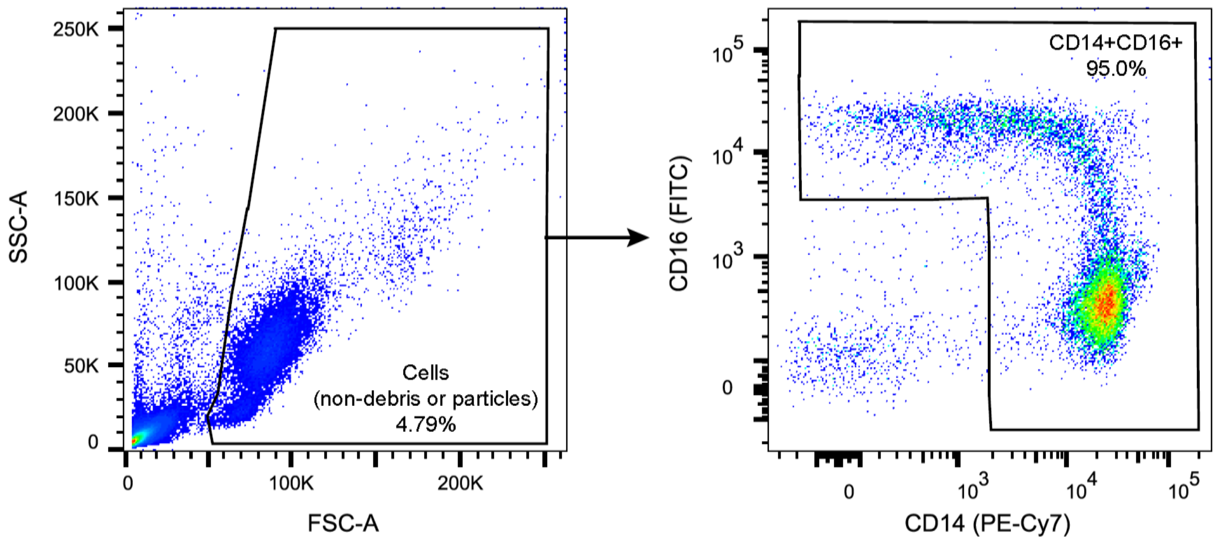

Supplementary Fig. 15. Gating for purity check upon purification of monocytes (CD14+/CD16+).

Uncropped gel for Supplementary Figure 5b

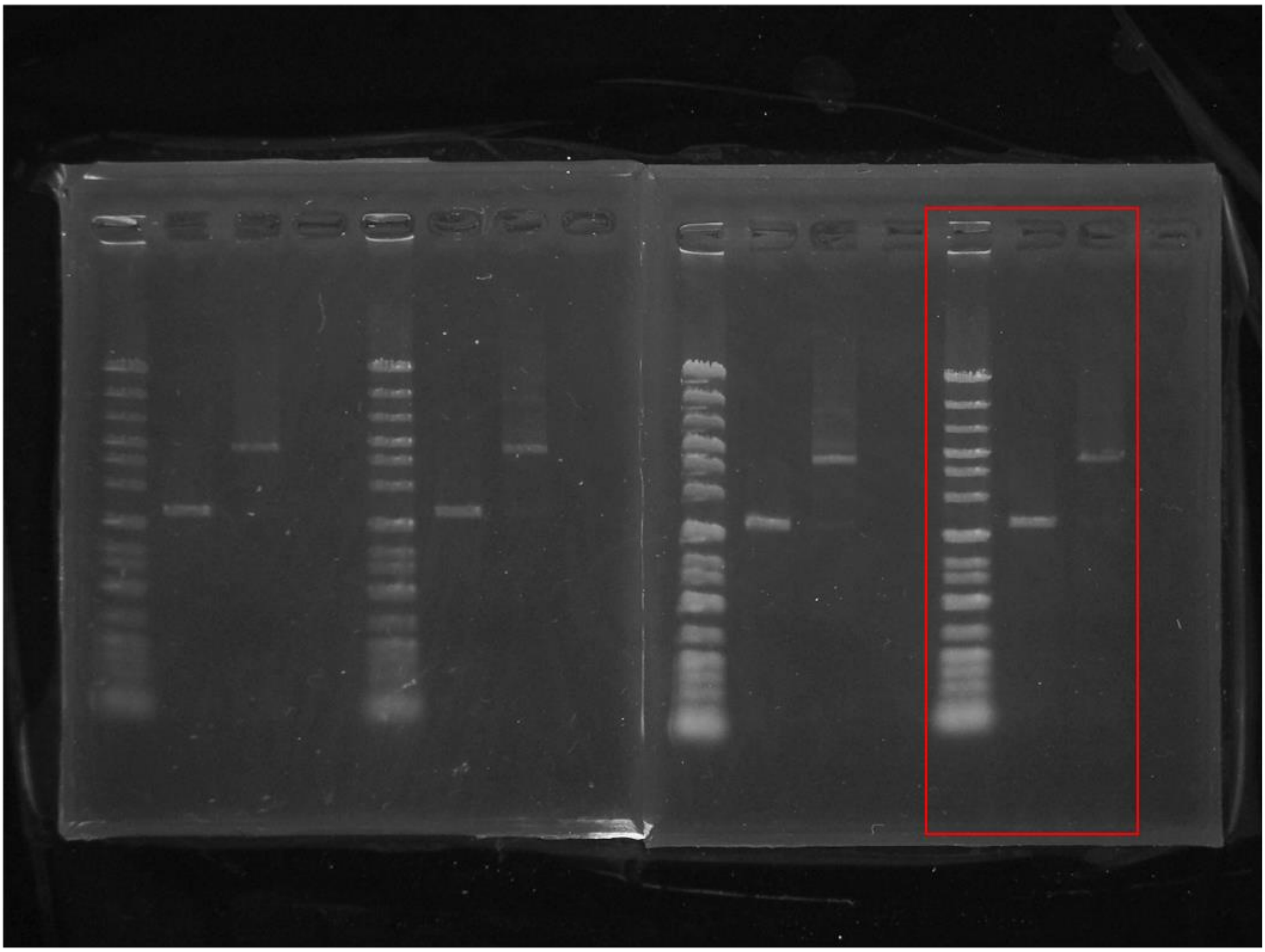

Uncropped gel for Supplementary Figure 7a

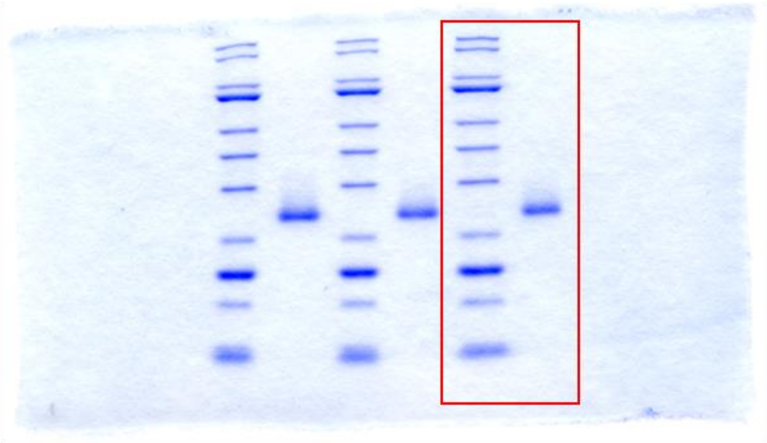

References

1. Laursen, M. F., Dalgaard, M. D. & Bahl, M. I. Genomic GC-Content Affects the Accuracy of 16S rRNA Gene Sequencing Based Microbial Profiling due to PCR Bias. *Front. Microbiol.* **8**, 1934 (2017).

2. Tulstrup, M. V. L. *et al.* Antibiotic Treatment Affects Intestinal Permeability and Gut Microbial Composition in Wistar Rats Dependent on Antibiotic Class. *PLoS One* **10**, (2015).

3. Frese, S. A. *et al.* Persistence of Supplemented Bifidobacterium longum subsp. infantis EVC001 in Breastfed Infants. *mSphere* **2**, (2017).

4. Lawley, B. *et al.* Differentiation of *Bifidobacterium longum* subspecies *longum* and *infantis* by quantitative PCR using functional gene targets. *PeerJ* **5**, e3375 (2017).

5. Matsuki, T. *et al.* Quantitative PCR with 16S rRNA-Gene-Targeted Species-Specific Primers for Analysis of Human Intestinal Bifidobacteria. *Appl. Environ. Microbiol.* **70**, 167–173 (2004).

6. Junick, J. & Blaut, M. Quantification of human fecal Bifidobacterium species by use of quantitative real-time PCR analysis targeting the groEL gene. *Appl. Environ. Microbiol.* **78**, 2613–2622 (2012).
