# Supplementary material for: Bifidobacterium species associated with breastfeeding produce aromatic lactic acids in the infant gut
Source: Nat Microbiol. 2021 Oct 21;6(11):1367–82. doi: 10.1038/s41564-021-00970-4 (PMC8556157; doi:10.1038/s41564-021-00970-4)
Supplement: Supplementary file 2 — Reporting Summary [file 41564_2021_970_MOESM2_ESM.pdf]

nature research

Corresponding author(s): Henrik Munch Roager
Tine Rask Licht

Last updated by author(s): Aug 23, 2021

Reporting Summary

Nature Research wishes to improve the reproducibility of the work that we publish. This form provides structure for consistency and transparency in reporting. For further information on Nature Research policies, see Authors & Referees and the Editorial Policy Checklist.

Statistics

For all statistical analyses, confirm that the following items are present in the figure legend, table legend, main text, or Methods section.

n/a	Confirmed	
☐	☒	The exact sample size (*n*) for each experimental group/condition, given as a discrete number and unit of measurement
☐	☒	A statement on whether measurements were taken from distinct samples or whether the same sample was measured repeatedly
☐	☒	The statistical test(s) used AND whether they are one- or two-sided<br>*Only common tests should be described solely by name; describe more complex techniques in the Methods section.*
☐	☒	A description of all covariates tested
☐	☒	A description of any assumptions or corrections, such as tests of normality and adjustment for multiple comparisons
☐	☒	A full description of the statistical parameters including central tendency (e.g. means) or other basic estimates (e.g. regression coefficient) AND variation (e.g. standard deviation) or associated estimates of uncertainty (e.g. confidence intervals)
☐	☒	For null hypothesis testing, the test statistic (e.g. *F*, *t*, *r*) with confidence intervals, effect sizes, degrees of freedom and *P* value noted<br>*Give P values as exact values whenever suitable.*
☒	☐	For Bayesian analysis, information on the choice of priors and Markov chain Monte Carlo settings
☒	☐	For hierarchical and complex designs, identification of the appropriate level for tests and full reporting of outcomes
☐	☒	Estimates of effect sizes (e.g. Cohen's *d*, Pearson's *r*), indicating how they were calculated

Our web collection on statistics for biologists contains articles on many of the points above.

Software and code

Policy information about availability of computer code

Data collection	No software was used
Data analysis	Quantification of aromatic amino acids in faeces and in vitro fermentation samples was performed using QuantAnalysis version 2.2 (Bruker Daltonics, Bremen, Germany). The raw urine metabolome data were converted to netCDF format using DataBridge Software version 3.5 (Waters, Manchester, UK) and imported into MZmine version 2.28. The raw untargeted faecal metabolome data were converted to mzXML files using Bruker Compass DataAnalysis 4.2 software (Bruker Daltonics). Gut microbiota data were processed by CLC Genomic Workbench (v8.5. CLCbio, Qiagen, Aarhus, DK), QIIME v1.967, and DADA2 pipeline (v.1.14). qPCR data were processed by the LightCycler® 480 Software v.1.5. Alignment of lactate dehydrogenase genes was constructed in CLC Main Workbench (v7.6.3, CLCbio, Qiagen, Aarhus, DK) and the phylogenic tree was visualized by use of the FigTree software v1.4.3 (http://tree.bio.ed.ac.uk/software/figtree/). The kinetic parameters (kcat, K0.5, and Hill coefficient nH) were calculated by curve-fitting the experimental data to the Hill equation, using GraphPad Prism v8.1. Bifidobacterium metagenome assembled genomes (MAGs) were identified by IGGsearch and IGGdb (v.1.0.087). Statistical analyses were performed using QIIME (v1.967), R (v3.189), GraphPad Prism (v8.1), and Matlab R2014b or 2015b (Mathworks, Inc.). The follow R packages were used: ggplot2 (v3.3.3), rmcorr (v0.4.3), maaslin2 (v1.0.0), gplots (v3.1.1)

For manuscripts utilizing custom algorithms or software that are central to the research but not yet described in published literature, software must be made available to editors/reviewers. We strongly encourage code deposition in a community repository (e.g. GitHub). See the Nature Research guidelines for submitting code & software for further information.

Data

Policy information about availability of data

All manuscripts must include a data availability statement. This statement should provide the following information, where applicable:

- Accession codes, unique identifiers, or web links for publicly available datasets
- A list of figures that have associated raw data
- A description of any restrictions on data availability

All 16S rRNA gene amplicon sequencing data were deposited in the Sequence Read Archive (SRA) under the BioProjects PRJNA273694 (SKOT) and PRJNA554596 (CIG). The following databases were used: GreenGenes 16S rRNA database (https://greengenes.secondgenome.com/), RDP database (https://rdp.cme.msu.edu/), 16S rRNA gene sequence database at NCBI (https://ncbiinsights.ncbi.nlm.nih.gov/2020/02/21/rrna-databases/), non-redundant protein sequence database at NCBI (https://www.ncbi.nlm.nih.gov/protein/), Swiss-Prot database (https://www.uniprot.org/), MBGD (Microbial Genome Database for Comparative Analysis; http://mbgd.genome.ad.jp/), and the genome database at NCBI (https://www.ncbi.nlm.nih.gov/genome/). Metabolomics data (concentrations of aromatic amino acid metabolites) from SKOT and CIG cohorts are available in Supplementary Data 1e and 2d. Source data are provided with this paper.

Field-specific reporting

Please select the one below that is the best fit for your research. If you are not sure, read the appropriate sections before making your selection.

☒ Life sciences ☐ Behavioural & social sciences ☐ Ecological, evolutionary & environmental sciences

For a reference copy of the document with all sections, see nature.com/documents/nr-reporting-summary-flat.pdf

Life sciences study design

All studies must disclose on these points even when the disclosure is negative.

Sample size	No sample size was estimated. Based on the original correlations observed between Bifidobacterium and aromatic lactic acids in 59 infants (SKOT), we estimated that 25 infants (CIG) with multiple time points would be sufficient to demonstrate the dynamics between Bifidobacterium species and aromatic lactic acids.
Data exclusions	In the CIG cohort, 6 samples were omitted from the Principal coordinate analyses due to low read counts (<8000). For correlation analyses between Bifidobacterium species and HMO residuals in faeces, 12 samples with no reported breastfeeding were excluded. One donor was excluded from the 200 µM indolelactate (ILA) stimulation of monocytes, since something went wrong during the stimulation.
Replication	All experiments were performed with full factorial (biological and technical) replication.
Randomization	No randomisation was performed as the study was observational.
Blinding	No blinding was performed as the study was observational.

Reporting for specific materials, systems and methods

We require information from authors about some types of materials, experimental systems and methods used in many studies. Here, indicate whether each material, system or method listed is relevant to your study. If you are not sure if a list item applies to your research, read the appropriate section before selecting a response.

Materials & experimental systems

n/a	Involved in the study	
☐	☒	Antibodies
☐	☒	Eukaryotic cell lines
☒	☐	Palaeontology
☐	☒	Animals and other organisms
☐	☒	Human research participants
☒	☐	Clinical data

Methods

n/a	Involved in the study	
☒	☐	ChIP-seq
☒	☐	Flow cytometry
☒	☐	MRI-based neuroimaging

Antibodies

Antibodies used	CD14-PE-Cy7 (eBiosciences, catalog number: 25-0149, clone name: 61D3) and CD16-FITC (Biolegend, catalog number: 302006, clone name: 3G8)
Validation	The 61D3 monoclonal antibody reacts with human CD14, a 53-55 kDa GPI-linked glycoprotein. CD14 is expressed on monocytes, interfollicular macrophages and some dendritic cells (manufacturer's website: https://www.thermofisher.com/antibody/product/CD14-Antibody-clone-61D3-Monoclonal/25-0149-42)

CD16 is known as low affinity IgG receptor III (FcγRIII). It is expressed as two distinct forms (CD16a and CD16b). CD16a (FcγRIIIA) is a 50-65 kD polypeptide-anchored transmembrane protein. It is expressed on the surface of NK cells, activated monocytes, macrophages, and placental trophoblasts in humans (manufacturer's website: https://www.biolegend.com/en-us/products/fitc-anti-human-cd16-antibody-567)

Eukaryotic cell lines

Policy information about cell lines

Cell line source(s)	Rat AhR Reporter Cells (H4IIE) was a personal gift from Dr. Michael S. Denison, Department of Environmental Toxicology, Meyer Hall, University of California, Davis California 95616, United States)<br>Human AhR Reporter Cells from Indigo Biosciences (PA, USA, catalogue number: IB06001)<br>cAMP Hunter™ eXpress GPR109B CHO-K1 GPCR Assay from DiscoverX (Fremont, CA, USA, catalogue number: 95-0141E2CP2M)
Authentication	None of the cells were tested for authentication.
Mycoplasma contamination	H4IIE cells were tested negative for mycoplasma contamination using the MycoAlert® Mycoplasma Detection Kit. The human Aryl Hydrocarbon Receptor (AhR) Reporter Assay System and the cAMP Hunter™ eXpress GPCR Assay were commercially available assays obtained from Indigo Biosciences and DiscoverX, respectively. We did not test these cells for mycoplasma contamination, but relied on the internal quality control of the manufacturers.
Commonly misidentified lines (See ICLAC register)	No commonly misidentified cell lines were used in the study.

Animals and other organisms

Policy information about studies involving animals; ARRIVE guidelines recommended for reporting animal research

Laboratory animals	Germ-free Swiss Webster mice (Tac:SW, originally obtained from Taconic Biosciences, NY, USA) were bred and housed at the National Food Institute, Technical University of Denmark. In two separate experiments, pregnant GF mice were randomised to be colonised with either B. longum 105-A wild-type (n=4) or aldh (type 4 ldh) mutant (n=5) by a single gavage one week prior to giving birth. The mono-colonised offspring (wildtype=21, 12 males and 9 females; aldh=29, 18 males and 11 females) were euthanised at 4 weeks of age.
Wild animals	No wild animals were used in the study.
Field-collected samples	No field-collected samples were used in the study.
Ethics oversight	All mouse experiments were approved by the Danish Animal Experiments Inspectorate (License number 2015-15-0201-00553) and carried out in accordance with existing Danish guidelines for experimental animal welfare.

Note that full information on the approval of the study protocol must also be provided in the manuscript.

Human research participants

Policy information about studies involving human research participants

Population characteristics	The discovery cohort consisted of a random subset of 59 healthy infants (30 male, 29 female) participating in the SKOT I study. Inclusion criteria were single birth and full term delivery, absence of chronic illness and age of 9 months ± 2 weeks at inclusion. The validation cohort, CIG, consisted of 25 healthy infants (12 male, 13 female), vaginally born (23/25) and full-term (23/25) delivered.
Recruitment	Infants in SKOT I were originally recruited by postal invitations to randomly selected parents of infants on the basis of extractions from the Danish National Civil Registration System. We used a random subset of this cohort for discovery.<br>For validating the results, infants in CIG were recruited through social media and limited to the Copenhagen region. These recruitments might have biased the recruitment towards an over-representation of breastfed, healthy infants from families with a high socioeconomic status. According to the GDPR rules, no information were available regarding covariate-relevant population characteristics of the healthy anonymous donor buffy coat material provided by the Blood bank at Rigshospitalet, Copenhagen. The only criteria was 'no intake of pain killers' on the day of blood draw.
Ethics oversight	The SKOT study protocol was approved by the Committees on Biomedical Research Ethics for the Capital Region of Denmark (H-KF-2007-0003) and The Data Protection Agency (2002-54-0938, 2007-54-026) approved the study.<br>The Committees on Biomedical Research Ethics for the Capital Region of Denmark confirmed that the CIG study was not notifiable according to the Act on Research Ethics Review of Health Research Projects (§ 1, subsection 4), as the study only concerned the faecal microbial composition and activity and not the health of the children. Informed consent was obtained from all parents of infants participating in the study. In addition, parents of CIG twins gave informed consent to publish data from the twins although the parents themselves would be able to identify their children using indirect identifiers. The Data Protection Agency (18/02459) approved the study. Use of the buffy coat material from healthy anonymous donors was approved by the Blood bank at Rigshospitalet, Copenhagen, under the jurisdiction of Region H.

Note that full information on the approval of the study protocol must also be provided in the manuscript.
